# Herlyn-Werner-Wunderlich Syndrome; laparoscopic treatment of obstructing longitudinal vaginal septum in patients with hematocolpos - a different technique for virgin patients

**DOI:** 10.4274/jtgga.galenos.2019.2019.0046

**Published:** 2020-12-04

**Authors:** Gökhan Boyraz, Alper Karalok, Taner Turan, Nejat Özgül

**Affiliations:** 1 Clinic of Obstetrics and Gynecology, University of Health Sciences Turkey, Ankara Etlik Zübeyde Hanım Women’s Health Training and Research Hospital, Ankara, Turkey

**Keywords:** Hematocolpos, longitudinal vaginal septum, Herlyn-Werner-Wunderlich Syndrome

## Abstract

We aimed to define a new laparoscopic treatment approach for patients with hematocolpos and obstructed hemi-vagina due to longitudinal obstructing vaginal septum. This technique is particularly useful for patients who desire to preserve virginity. To the best of our knowledge this is the first case reporting laparoscopic resection of vaginal septum with an obstructed hemivagina and hematocolpos.

## Introduction

Herlyn-Werner-Wunderlich Syndrome is a rare congenital anomaly characterized by uterus didelphys with blind hemivagina and ipsilateral renal agenesis and was initially described by Herlyn and Werner in 1971. The true incidence of this anomaly is unknown, however it has been reported between 0.1% and 3.8% (1,2).

**Figure 1:**
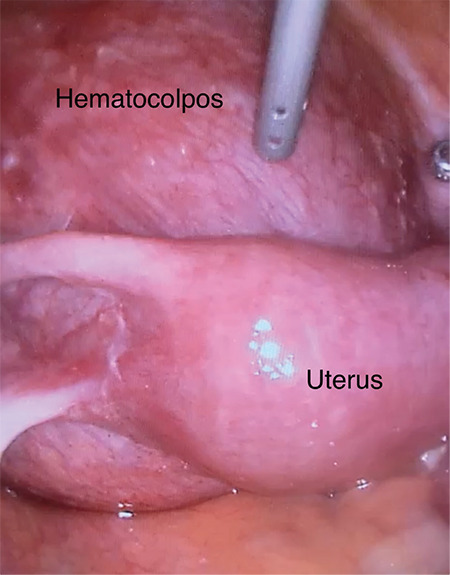
A wide hematocolpos corresponding to the obstructed left hemivagina

**Figure 2:**
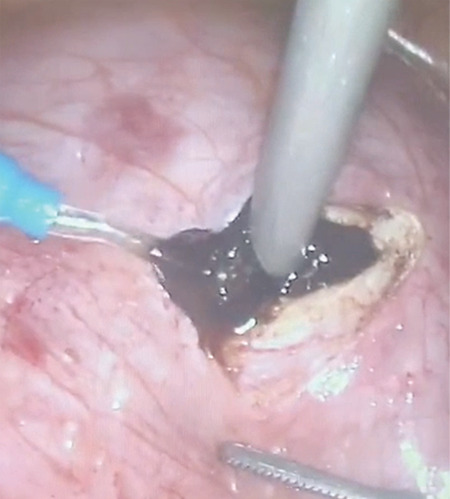
Draining the old menstrual blood

**Figure 3:**
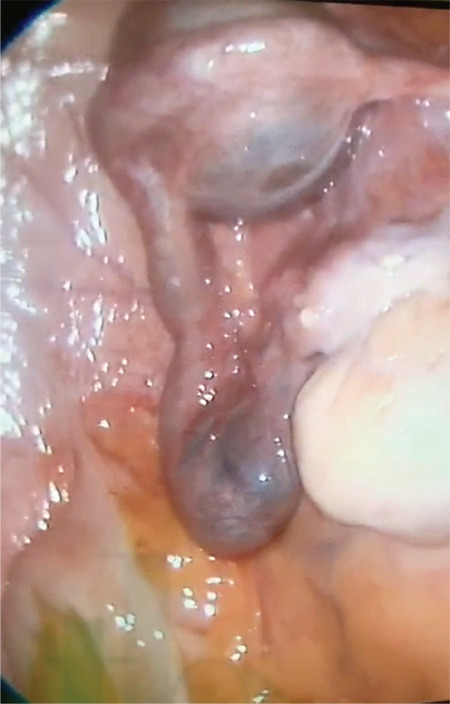
Fimbrial phimosis of left fallopian tube

A 30-year-old patient presented with severe abdominal-pelvic pain and dysmenorrhea. Pelvic magnetic resonance imaging indicated a complete uterine septum coexisting with longitudinal obstructing vaginal septum that might cause hematocolpos. Unilateral renal agenesis was detected in computerized tomography urogram. She had not been sexually active and in spite of the severe pelvic pain she absolutely rejected vaginal surgery in order to preserve her hymeneal integrity and virginity. This situation forced the use of a laparoscopic approach. Therefore, we aimed to define a new laparoscopic treatment approach for the patients with hematocolpos and obstructed hemi-vagina due to longitudinal obstructing vaginal septum. This technique is particularly useful for patients who desire to preserve virginity. All of the techniques described previously were based on a vaginal approach and, to the best of our knowledge, this is the first case reporting laparoscopic resection of vaginal septum with an obstructed hemivagina and hematocolpos. This laparoscopic approach in patients with obstructing longitudinal vaginal septum with hematocolpos not only preserves hymeneal integrity but also enables definition of genital tract anomalies and coexisting anomalies exactly. The procedure consisted of two major steps (Video 1). Firstly, a transverse incision is made in the anterior vagina wall (Figure 1). Secondly, the longitudinal vaginal septum is resected (Figure 2) and transverse vaginal incision is closed with intra-corporeal suturing (Figure 3).

## Conclusion

This first description of a laparoscopic approach seems to be an alternative treatment options in patients with hematocolpos, especially in those who desire to preserve virginity.

**Video 1. https://www.doi.org/10.4274/jtgga.galenos.2019.2019.0046.video1**
